# Genome-wide linkage analysis of blood pressure under locus heterogeneity

**DOI:** 10.1186/1471-2156-4-S1-S78

**Published:** 2003-12-31

**Authors:** Xinqun Yang, Kai Wang, Jian Huang, Veronica J Vieland

**Affiliations:** 1 Department of Biostatistics, Division of Statistical Genetics, The University of Iowa, Iowa City, USA; 2 Department of Statistics and Actuarial Science, The University of Iowa, Iowa City, USA; 3 Department of Psychiatry, The University of Iowa, Iowa City, USA

## Abstract

We describe a method for mapping quantitative trait loci that allows for locus heterogeneity. A genome-wide linkage analysis of blood pressure was performed using sib-pair data from the Framingham Heart Study. Evidence of linkage was found on four markers (GATA89G08, GATA23D06, GATA14E09, and 049xd2) at a significance level of 0.01. Two of them (GATA14E09 and 049xd2) seem to overlap with linkage signals reported previously, while the other two are not linked to any known signals.

## Background

High blood pressure (BP) is an important risk factor for cardiovascular disease and is a leading cause of mortality in industrialized countries [1]. Being a complex trait, BP may be influenced by genes at different locations on the genome in different families (inter-family locus heterogeneity). Presumably, allowing for locus heterogeneity in the analysis will increase the power to detect linkage.

We propose a model that allows for locus heterogeneity for quantitative trait loci (QTLs). This model generalizes the locus heterogeneity model for qualitative traits [2,3] to the case of continuous traits. A genome-wide linkage analysis was conducted on the Framingham Heart Study data using the likelihood-ratio statistic based on this model.

## Methods

### Data

The Framingham Heart Study consists of two cohorts with a total of 10,333 subjects. The first cohort includes 5209 individuals who were recruited initially in 1948 when the study began. The second cohort includes 5124 subjects who are offspring, or spouses of offspring, of the initial participants. Longitudinal data were recorded over follow-up years in both cohorts. In the total 330 pedigrees provided by the Genetic Analysis Workshop 13 (GAW13), 1702 subjects were genotyped at at least one marker and 2885 subjects were phenotyped for BP at least once.

To remove the effect of hypertension treatment, we focused only on those subjects who never received any antihypertensive medication. There are 1909 such subjects. Their characteristics are summarized in Table 1. The systolic blood pressures were adjusted for age, sex, and body mass index (BMI) in the following manner. The averages of BP, age, and BMI were computed for each subject, and then the average of BP was regressed over those of age and BMI as well as sex, separately for each of the two cohorts. The residuals of the simple linear regression were used as the (adjusted) phenotypic values. The two-point identity-by-descent (IBD) sharing probabilities on relative pairs provided by GAW13 were used. Only sib pairs were used in the analysis. Sib pairs from the same sibship or from different sibships in the same pedigree were treated as if they were biologically unrelated.

A total of 400 markers were used in the genome scan. Not all sib pairs were genotyped at every marker. So the number of sib pairs used in the analysis varied from marker to marker, roughly from 400 to 1500.

Significant departure from normality was detected on the (adjusted) BP measurement using the Shapiro-Wilk test (*p*-value < 0.0001). To reduce the impact of the non-normality of the data on the performance of the proposed statistic, the BP measurements were transformed based on the normal copula model [4] so the transformed trait values follow a standard normal distribution, with a mean and variance of 0 and 1, respectively.

### Statistical methods

Consider a sib pair whose trait values are denoted by y_1 _and y_2_, respectively. The trait values are standardized such that they have mean 0 and variance 1. It is assumed that the candidate locus has an additive effect but no dominance effect on the trait. Let ρ_0_, ρ_1_, and ρ_2 _be the correlation coefficients of y_1 _and y_2 _when the number of alleles that are shared IBD by the sib pair is 0, 1, and 2, respectively. Because there is no additive effect, we have ρ_1 _= (ρ_0 _+ ρ_2_)/2 [4]. When there is no linkage, ρ_1 _can be estimated by taking the value of correlation coefficient between y_1 _and y_2 _[5,6]. So ρ_1 _can be treated as known.

Conditional on the IBD sharing status, *y*_1 _and *y*_2 _are assumed to have a bivariate normal distribution, which is completely characterized by the correlation coefficients ρ_0_, ρ_1_, or ρ_2_. Let π_0_, π_1_, and π_2 _be the probabilities that the sib pair shares 0, 1, or 2 alleles IBD, respectively, and then the likelihood function for *y*_1 _and *y*_2 _is

L(ρ_2_; y_1_, y_2_) = π_0 _φ (y_1_, y_2_; 2ρ_1 _- ρ_2_) + π_1 _φ (y_1_, y_2_; ρ_1_) + π_2 _φ (y_1_, y_2_; ρ_2_),

where φ(·,·;·) denotes the density function of a bivariate normal distribution. When the candidate locus is not linked to any QTL, ρ_1 _= ρ_2 _and the above likelihood reduces to *L*(ρ_1_; *y*_1_,*y*_2_) = φ(*y*_1_, *y*_2_; ρ_1_). Otherwise, ρ_2 _> ρ_1_.

Let α be the probability that a QTL is linked to the candidate locus for the sib pair. Then the likelihood function for the sib pair would be

α L(ρ_2_; y_1_, y_2_) + (1 - α)L(ρ_1_; y_1_, y_2_).

Let *i *index the *i*^th ^sib pair in a collection of sib pairs, then the log-likelihood function for these sib pairs is

l(α, ρ_2_) = Σ_i _log[α L(ρ_2_; y_i1_, y_i2_) + (1 - α)L(ρ_1_; y_i1_, y_i2_) ],

where *y*_i1 _and *y*_i2 _are the trait values for the *i*^th ^sib pair. The hypotheses of interest are

*H*_0_: ρ_2 _= ρ_1_, α ∈ [0,1] vs. *H*_1_: ρ_2 _> ρ_1_, α ∈ [0,1]

This model is an extension of the heterogeneity model of Smith [2] for dichotomous traits to continuous traits. The likelihood ratio statistic was used as the test statistic. To obtain the maximum likelihood estimates (MLEs) of parameters, we used a two-dimensional grid search over α and ρ_2_, with the grid size of 0.01 for both parameters.

The asymptotic distribution of the likelihood ratio statistic is not known analytically. So a simulation study with 10,000 replicates was carried out. The trait locus is assumed to have two equally frequent alleles and its heritability is set to 0.3. The unlinked marker is assumed to be fully polymorphic. The 90^th^, 95^th^, 99^th^, and 99.9^th ^percentiles of the distribution are found to be 2.72, 3.91, 6.61, and 11.07, respectively.

## Results

A genome scan is conducted using three statistics: the test statistic of Haseman-Elston method (H-E) [7], the likelihood ratio statistic (HET-LRT) for the heterogeneity model described above, and the likelihood ratio statistic (HOM-LRT) for the homogeneity model, which is a special case of the heterogeneity model with α fixed at 1. The markers that are significant for at least one statistic at significance level 0.01 are listed in Table 2.

At significance level 0.01, four significant markers are identified by statistic HET-LRT. Among the four markers, GATA14E09 on chromosome 8 is very close to 8q21.11, a region that showed linkage to BP [8]. 049xd2 on chromosome 16 is between 16p12 and 16p13.1, a region in which linkage to BP was found by two independent studies [8,9]. To the best of our knowledge, the other two markers, i.e., GATA89G08 on chromosome 5 and GATA23D06 on chromosome 8, do not link to any previous findings.

## Discussion

We introduce a heterogeneity model for mapping QTLs using sib-pair data. A genome scan was performed to map QTLs affecting BP variation on a population-based data using this new method. At significance level 0.01, evidence of linkage is found in four marker regions on chromosomes 5, 8, and 16. Two of the markers seem to overlap with linkage signals in previous studies, while the other two are not linked to any previous findings.

Of the three statistics used in the genome scan, H-E provides the most linkage signals, followed by HOM-LRT, and then by HET-LRT. At the four markers at which HET-LRT is significant (and at many nonsignificant markers that are not shown), the statistic value of HET-LRT is the same as that of HOM-LRT. Finer grid size may change the values of HOM-LRT and HET-LRT, but the changes are expected to be small. Experiments on the four significant markers with grid size 0.001 strongly support this claim. In these experiments, HET-LRT is still the same as HOM-LRT for all four markers.

The proposed statistic HET-LRT is intended for a population sample. Its performance under selected samples is unknown. Although the normal copula model can be used on selected samples to recover normality, its effectiveness has yet to be investigated. It is worthwhile to point out that our analysis has excluded individuals on antihypertensive medication. Therefore, careful attention should be paid when generalizing our results to general population. For a recent review on the issues dealing with antihypertensive treatments, see Palmer [10].

We have been using the posterior probability of linkage (PPL) to assess linkage signals across heterogeneous data sets [11-14]. Since the proposed heterogeneity model for quantitative traits is parallel to that for qualitative traits, it is reasonable to adapt our previous work on qualitative traits to the current setting. The details are being worked out.

The proposed model is for sib-pair data only. It is expected that the use of nonindependent sib-pairs, like what we did in this analysis, does not affect the type I error rate asymptotically. It is of interest to see how this model can be generalized to general pedigrees and how the generalized model performs.

## Figures and Tables

**Table 1:** Summary statistics for subjects in Cohort 1 and Cohort 2 (standard errors are in parentheses)

	**Number by Gender**					
				
**Cohort**	**Male**	**Female**	**Total**	**Age (yr)**	**BMI (kg/m^2^)**	**SBP (mm Hg)**
1	304	269	573	56.0 (7.4)	25.7 (3.7)	130.1 (16.1)
2	659	677	1336	40.9 (10.1)	25.5 (4.3)	118.5 (12.3)

**Table 2:** Significant markers at significance level 0.01 (*p*-values are in parentheses)

**Marker**	**Chr**	**cM**	**H-E**	**HOM-LRT**	**HET-LRT**
GATA6F06	3	79	5.40 (0.009)	6.02 (0.007)	
GATA28F03	4	73	7.42 (0.003)		
GATA89G08	5	98	10.06 (0.001)	6.68 (0.005)	6.68 (0.009)
GATA23D06	8	26	6.99 (0.004)	6.68 (0.005)	6.68 (0.009)
GGAA20C10	8	60	6.45 (0.005)		
GATA14E09	8	94	11.36 (0.0003)	6.76 (0.004)	6.76 (0.009)
GATA21C12	8	140	6.15 (0.006)		
GATA62F03	9	14	10.88 (0.0004)		
GGAA2F11	10	117	8.99 (0.001)	5.42 (0.010)	
ATA29A06	12	161	10.79 (0.0004)		
GATA13D05	12	166	6.50 (0.005)		
GATA30A03	14	92	9.38 (0.001)		
ATA3A07	16	23	7.64 (0.002)	5.39 (0.010)	
049xd2	16	44	8.92 (0.001)	7.23 (0.003)	7.23 (0.007)
ATC6A06	17	67	5.50 (0.009)		
ATA7D07	18	89	6.81 (0.004)		
GATA188F04	21	40	6.05 (0.006)	5.88 (0.008)	
